# COVID-19 Infection and Acute Myeloid Leukemia: A Likely Marriage?

**DOI:** 10.7759/cureus.72940

**Published:** 2024-11-03

**Authors:** Younus Al-Midfai, Moshe Bengio, Pallavi Aneja

**Affiliations:** 1 Internal Medicine, HCA Healthcare Westside Hospital, Plantation, USA; 2 Internal Medicine, Northwest Medical Center, Margate, USA; 3 Medical School, Nova Southeastern University Dr. Kiran C. Patel College of Osteopathic Medicine, Davie, USA; 4 Emergency Medical Services, Hatzalah South Florida EMS, Miami, USA; 5 Emergency Medicine, HCA Florida Aventura Hospital, Aventura, USA; 6 Internal Medicine, HCA Florida Westside Hospital, Plantation, USA

**Keywords:** acute myeloid leukemia, covid-19, internal medicine, leukocytosis, myeloproliferative disorder

## Abstract

In the early era of COVID-19, characterization of long-term complications was unfeasible. But in early 2021, the authors came across a patient with a new diagnosis of Acute Myeloid Leukemia (AML) that was highly suspected as being secondary to a previous COVID-19 infection. The similarities of this anomaly to Hodgkin Lymphoma occurring after Epstein Barr Virus prompted the authors to perform a qualitative review of the literature for a possible pathway of how COVID-19 can cause AML.

## Introduction

The SARS-CoV-2 virus (that causes coronavirus disease 2019, COVID-19) is in a state of flux. On one hand, researchers have learned a great deal about active COVID-19 disease. On the other, some research has been published on post-viral adverse effects and little research is available on COVID-19 as a propagator of other illnesses, like cancer, in the very long term.

To put this article in the correct context, the initial draft of this article was presented as a poster abstract in the summer of 2021 during the height of the COVID-19 pandemic (Poster Abstract: Al-Midfai Y., Bengio M., Aneja P. COVID-19 as a Potential Pathogenic Trigger for Acute Myeloid Leukemia (AML). Westside Regional Medical Center Graduate Medical Education Poster Competition, June 2021). At this conference, the authors received mixed reviews from the attendees with the majority of physicians of advanced specialties negating our theoretical conclusion as not possible. We presume the reason for this was the fear of having a major pandemic virus causing malignancy; therefore, we felt our article was not appropriate to publicly publish at that time. Fast forwarding to the time of this publication during the summer of 2024, more data, as will be highlighted in the upcoming paragraphs, has become available on COVID-19, with discussions on its long-term effects.

COVID-19 has already evolved many times with highs and lows in severity. Unlike influenza or RSV, the peaks could not be predicted during the winter season and tend to peak randomly within multiple seasons. COVID-19 is further complicated by its rapidly evolving nature, leading to the population's inability to form an immunity to the virus despite vaccine use [1].

On an individual level, comorbid conditions were found to also complicate the course of the disease despite the low severity of the reigning COVID-19 strain in healthy individuals at any certain time. Age is a major factor, with those 65 years or older 97 times more likely to have a fatal outcome than those between 18 and 27 years of age [2]. Other factors which are flagged by the Centers for Disease Control and Prevention (CDC) as high risk include cancers, cerebrovascular disease, chronic renal, liver, or lung diseases, cystic fibrosis, dementia, diabetes types I and II, heart disease, hemoglobinopathies, HIV, Immunocompromising state, obesity, sedentary lifestyle, pregnancy, smoking, and substance abuse [2]. The list is extensive and is not all-inclusive. However, the common denominator of these comorbidities and fluctuations is the fact that they have occurred since the onset of the COVID-19 pandemic until now and have been studied and analyzed retrospectively by many organizations, like the CDC and National Respiratory and Enteric Virus Surveillance System (NREVSS).

When turning our eye to the future, there have been some studies that follow those who recovered from COVID-19. A 2021 article* *reported a systematic review of 21 meta-analyses encompassing 47,910 patients between ages 17 and 87 reported persistence of at least one symptom from among 55 possible symptoms in 80% of those who previously had COVID-19 two weeks or more prior, with fatigue and headache most commonly persistent in around 50% of individuals [3]. In 2023, *Nature Review Microbiology *published a new article on guidelines and recommendations on what has become known as long COVID with preliminary data showing 65 million people affected worldwide, though likely significantly underestimated. In these patients, 200 possible symptoms were identified. Long COVID is a developing area of research but hypotheses in its pathogenicity revolve around COVID-19 altering many physiologic processes and creating the groundwork for the persistence of or development of the various symptoms [4]. The ability of COVID-19 to cause these changes makes one wonder about the extent of COVID-19 adversity.

The concept of COVID-19 as an oncogenic consideration has not been proven but remains a concerning area that may yet be too premature to quantitatively analyze at around four years since the onset of COVID-19. However, it is important to be aware of the possibilities. This article will begin with a case presentation of a new diagnosis of AML and then discuss its theorized relationship to a previous COVID-19 infection. 

## Case presentation

A 53-year-old male with a past medical history of COVID-19 infection requiring O2 therapy via non-rebreather during the previous hospitalization nine months before current hospital admission currently presents with fatigue, generalized weakness, and a left shoulder abscess. The patient states that the fatigue and weakness started about one week ago, but he has been dealing with chronic abscess formation for the last three months. The abscesses arise after abrasions or bruising. Two prior abscesses have since healed. The patient also admits fever, chills, shortness of breath, and 50-pound weight loss over the past nine months, with two episodes of sudden onset nausea and vomiting at work over the last few months. He denies chest pain, abdominal pain, headache, frequency, urgency, constipation, and diarrhea.

The patient’s medical history includes pre-diabetes, nephrolithiasis, and wrist surgery. He takes naproxen at home for pain and has no allergies. He has never had a colonoscopy. He admits to marijuana use but denies any tobacco or alcohol. 

Upon presentation, the vital signs were 36.7 ℃ temperature, heart rate of 88 beats/minute, respiratory rate of 19 breaths/minute, blood pressure of 127/80 mmHg, and 100% O2 saturation on room air. The pertinent labs are in Table 1. The patient was overall unremarkable on the physical exam except for a left shoulder abscess that was drained and dressed in the emergency department and multiple healed ulcers.

**Table 1:** Pertinent Lab Values Table 1 displays the patient's pertinent laboratory findings compared to the normal values

Pertinent labs	Value	Normal
White Blood Cell	75 x 10^3/µL with left shift	4.0–10.0 x 103/µL
Hemoglobin	7.0 g/dL	14–17 g/dL
Platelets	54/µL	150,000–350,000/µL
Creatinine	1.16 mg/dL	0.7–1.3 mg/dL
Glucose	144 mg/dL	70–100 mg/dL

Due to the leukocytosis, the patient was a candidate for a bone marrow biopsy, which showed 20% blasts consistent with acute myeloid leukemia (AML). Regrettably, the pathology images were not attainable due to departmental policy. The abscess was treated with ceftriaxone, vancomycin, and flagyl. The AML treatment course per hematology/oncology consultation was a 7+3 chemotherapy protocol with idarubicine and cytarabine. Following up on day 11 of admission/day 6 of chemotherapy, the labs showed 0.9/u leukocytes, pancytopenia, and hemoglobin of 7.7 g/dL.

Due to the sequence of events, the researchers suspect that the first symptom of AML, which was weight loss, had begun because of COVID-19 infection.

## Discussion

The concept of post-recovery malignant precipitation is not yet well-researched for COVID-19 but is not foreign to other microbial infections. Hjalgrim, et al. is one of many studies to report the relationship between EBV and certain lymphomas. The researchers estimated that at least one patient would contract Hodgkin Lymphoma for every 1000 individuals previously infected with Epstein-Barr virus (EBV) [5]. The exact mechanism is unknown, and the reports on the EBV episome’s proposed impact on triggering lymphoma by remaining latent inside previously infected B lymphocytes are beyond this report's scope [6]. However, there are reports that this process takes approximately four to five years post-infection, with the peak risk being as early as 2.4 years [5,7].

Regarding AML, previous studies have shown that most AML cases are likely a result of prior infections or autoimmune disease; only a small minority are due to radiation, chemical exposures, chemotherapy, myeloproliferative neoplasms, or Down Syndrome [8]. Recent literature on SARS-CoV-2 proposed infiltration of host cells suggests that the SARS-CoV-2 S spike protein binds the surface protein angiotensin-converting enzyme 2 (ACE2), which lung and intestinal epithelial cells express, in addition to hematopoietic stem/progenitor cells (HSPCs) [9]. After the internalization of ACE2 during viral entry, the proteins that commonly bind ACE2, like angiotensin II, accumulate and instead bind the angiotensin II (AT1) receptors expressed in the same organ systems [10,11]. Activating AT1 and potentially ACE2 (and another pathway, ComC (complement cascade)) directly activates the Nlrp3 inflammasome, a robust pro-inflammatory organelle. This process likely leads to a cytokine storm [12].

Additionally, Ratajczak, et al. identified the Nlrp3 inflammasome as a potent malignant factor that causes damage to HSPCs. Hence, with the evidence that the COVID-19 cytokine storm activates the Nlpr3 inflammasome, it would seem logical to also set in motion events that may lead to malignant processes [12]. If this is the case, then proposed COVID-19 treatments, like Quercetin, an Nlrp3 inflammasome inhibitor, could treat acute COVID-19 infections by inhibiting the cytokine storm and, ultimately, prevent the formation of malignant processes [13]. Despite the above proposals on COVID-19 mechanisms, the studies are still qualitative and theoretical. Therefore, before a qualitative study could in any way validate the above-proposed mechanisms, the question remains: Will future quantitative analyses concur that COVID-19 is correlated with malignant processes like AML?

## Conclusions

We found a link that connected our patient’s current presentation of AML with his past medical history of COVID-19. Our findings implicated the involvement of HSPCs in the acute cytokine storm and the long-term effect of leukemia. We hope this report will open doors to further research into the HSPC’s participation in the cytokine storm and hematological processes, in addition to long-term quantitative studies on the relationship between COVID-19 and AML.
